# High Public-Health Impact in an Influenza-B-Mismatch Season in Southern Italy, 2017-2018

**DOI:** 10.1155/2019/4643260

**Published:** 2019-08-20

**Authors:** Daniela Loconsole, Anna Lisa De Robertis, Anna Morea, Daniele Casulli, Rosanna Mallamaci, Simona Baldacci, Francesca Centrone, Viviana Bruno, Michele Quarto, Marisa Accogli, Maria Chironna

**Affiliations:** ^1^Department of Biomedical Sciences and Human Oncology-Hygiene Section, University of Bari, Piazza G. Cesare 11, 70124 Bari, Italy; ^2^Department of Biosciences, Biotechnology and Biopharmaceutics, University of Bari, Via Orabona 4, 70124 Bari, Italy

## Abstract

**Background:**

Yearly influenza epidemics have considerable effects on public health worldwide. The 2017-2018 influenza season in Italy was of greater severity than previous seasons. The aim of this study was to describe the 2017-2018 influenza season in Southern Italy and the molecular characteristics of the circulating viral strains.

**Methods:**

The incidence of influenza-like illness (ILI) was analysed. Nasopharyngeal swabs collected from patients with ILI from week 46/2017 to week 17/2018 were tested to identify influenza A viruses (IAV) and influenza B viruses (IBV). Sequencing and phylogenetic analysis of haemagglutinin genes were also performed on 73 positive samples (35 IBV, 36 IAV H1, and 2 IAV H3 strains).

**Results:**

During the 2017-2018 season, the peak incidence was 14.32 cases per 1,000 inhabitants. IBV strains were identified in 71.0% of cases. The 35 characterised IBV strains belonged to Yamagata lineage clade 3, the 36 A/H1N1pdm09 strains clustered with the genetic subgroup 6B.1, and the 2 A/H3N2 strains clustered with the genetic subgroup 3C.2a. Intensive-care unit (ICU) admission was required in 50 cases of acute respiratory distress syndrome (ARDS). Among the >64-year age group, 18 out of 26 ICU-ARDS cases (69.2%) were caused by IBV, and 14 of these (77.8%) were B/Yamagata lineage.

**Conclusions:**

The 2017-2018 influenza season was one of the most severe in a decade in Southern Italy. IBV mismatch between the trivalent vaccine and the circulating strains occurred. The high number of ICU-ARDS cases caused by B/Yamagata strains in the >64-year age group suggests that further data on the effectiveness of the available influenza vaccines are needed to determine the best way to protect the elderly against both IBV lineages.

## 1. Introduction

Yearly influenza epidemics have severe effects on public health worldwide. Influenza can lead to hospitalisation and death, especially in the elderly and in individuals with comorbidities [1]. The annual attack rate is estimated to reach 5–10% in adults and 20–30% in children [2]. Every year, 3–5 million cases of severe influenza and around 290,000–650,000 influenza-related deaths occur [3].

Vaccination is the most effective way to prevent influenza virus infection and its complications. However, because of antigenic drift in seasonal influenza viruses, the composition of the vaccines must be changed every year. In Italy, the recommendations for the use of the available influenza vaccines are reported yearly in a document of the Ministry of Health. For the 2017-2018 influenza season, for the northern hemisphere, the recommended vaccine formulation for the trivalent vaccines was an A/Michigan/45/2015 (H1N1)pdm09-like virus, an A/Hong Kong/4801/2014 (H3N2)-like virus, and a B/Brisbane/60/2008-like virus. The quadrivalent vaccine also contained a B/Phuket/3073/2013-like virus [4].

Global surveillance is the main tool for monitoring influenza. In 2011, the World Health Organization Global Influenza Surveillance Network (WHO GISN) was converted into the WHO Global Influenza Surveillance and Response System (WHO GISRS) [5]. In the WHO European Region, the national sentinel surveillance system covers from 1% to 5% of the population (depending on the country) and is based on a network of primary care physicians who report the weekly number of influenza-like illnesses (ILIs) and/or acute respiratory infections (ARIs) to the national focal point for influenza surveillance [6]. From week 40 of one year to week 20 of the following year, data are reported weekly to the European Centre for Disease Prevention and Control (ECDC) through the European Surveillance System (TESSy) [6]. In Italy, paediatricians and general practitioners (GPs) of the national InfluNet network report the ILIs (epidemiological surveillance) and collect respiratory specimens to identify the circulating influenza strains (virological surveillance) [7].

In Italy, the estimated incidence of influenza in the 2017-2018 season was >8.5 million [8], which indicates greater severity than previous seasons. The epidemic period started earlier than usual, and the peak incidence (estimated at 14.73 per 1,000 inhabitants) was observed in the week 02/2018 [8]. Some 60% of all laboratory-confirmed influenza cases were caused by influenza B virus (IBV) [9].

No recent data are available on the epidemiology of influenza and on the circulation of influenza viruses during recent seasons in Southern Italy. The aim of this study was to describe the 2017-2018 influenza season in the Apulia region (Southern Italy), along with the molecular characteristics of the circulating strains.

## 2. Material and Methods

### 2.1. Study Population

The Apulia region has a population of >4 million people. According to the national protocol, the regional influenza surveillance system surveys about 2% of the general population (80,000 inhabitants) and is based on a sentinel-physician network of paediatricians and GPs who report ILIs weekly [7]. Epidemiological and virological surveillance are managed by the Apulian Regional Observatory for Epidemiology. ILI is defined as a sudden onset of symptoms, with at least one of four systemic symptoms (fever or feverishness, malaise, headache, and myalgia) and at least one of three respiratory symptoms (cough, sore throat, and shortness of breath) [10]. For epidemiological purposes, the number of reported ILIs from week 42/2017 to week 17/2018 was analysed. For virological purposes, nasopharyngeal swabs were collected by sentinel physicians from nonhospitalised patients with ILIs from week 46/2017 to week 17/2018. Respiratory samples such as nasopharyngeal swabs, bronchoalveolar lavages, and bronchial aspirates were also collected from throughout the region from hospitalised patients with severe acute respiratory illness (SARI), defined as fever and at least one sign or symptom of acute respiratory illness (cough, sore throat, shortness of breath, and coryza) [10], and from patients admitted to all intensive-care units (ICUs) of the region with acute respiratory distress syndrome (ARDS), during the influenza season.

All the samples were analysed at the Laboratory of Molecular Epidemiology and Public Health of the Hygiene Unit of Policlinico Hospital Bari, which is the Regional Reference Laboratory for the virological surveillance of influenza. All procedures performed in the study were in accordance with the ethical standards of the institutional and national research committee and with the 1964 Helsinki declaration and its later amendments or comparable ethical standards. Ethical approval was obtained from the Institutional Review Board at the Apulian Regional Observatory for Epidemiology. Informed written consent was obtained from all individuals or legal guardians of the children who provided the specimens. Statistical analysis was performed using STATA 12.0 software. The Chi-squared or Fisher's exact test were used to compare proportions. A* p value* of <0.05 was considered significant.

### 2.2. Influenza Detection and Subtyping

Respiratory samples were subjected to nucleic acid extraction by MagNA Pure, according to the manufacturer's instructions (Roche Diagnostics, Mannheim, Germany). Influenza viruses were detected with a commercial real-time PCR assay (Anyplex RV16 Detection; Seegene, Seoul, Republic of Korea). Subtyping of influenza A virus (IAV) was performed with a commercial real-time PCR assay (FTD Flu differentiation; Fast Track Diagnostics, Esch-sur-Alzette, Luxembourg). Specimens that were positive for IBV were subtyped with a reverse-transcription PCR (RT-PCR) assay targeting the specific HA genes of IBV Victoria and Yamagata lineages. The assay was performed with primers and conditions previously reported by the WHO [11].

### 2.3. Sequencing and Phylogenetic Analyses

A series of positive samples (*n* = 73) was collected by convenience sampling from cases identified through InfluNet surveillance of individuals with ILI and from patients with SARI or ICU-ARDS, and was subjected to sequencing and phylogenetic analyses. Sequencing in the HA gene was performed using conventional Sanger-sequencing methodology: 35 were IBV (four from individuals with ILI, 17 from patients with SARI, and 14 from ICU-ARDS), 36 were influenza A/H1N1pdm09 (five from individuals with ILIs, 10 from patients with SARI, and 21 from ICU-ARDS), and two were influenza A/H3N2 (one from a patient with SARI and one from ICU-ARDS). The SuperScript III One-Step PCR Amplification Kit (Invitrogen, Monza, Italy) was used for HA gene amplification according to the WHO protocol [11], and PCR products were purified with a PCR purification kit (Qiagen, Milano, Italy). Sequences were assembled and analysed with CLC DNA Workbench 6.0 (Qiagen). Molecular Evolutionary Genetics Analysis (Mega) 7 software (https://www.megasoftware.net/) was used for sequence alignment by the ClustalW method, for comparison of sequences with those contained in the GenBank and Global Initiative on Sharing Avian Influenza Data (GISAID) databases, and for generation of phylogenetic trees. Most of the sequences generated in this study were deposited in the GISAID database. Sequence accession numbers are reported in the phylogenetic trees. No depositing in GISAID was made for sequences nearly identical to that already deposited.

## 3. Results

**Figure 1:**
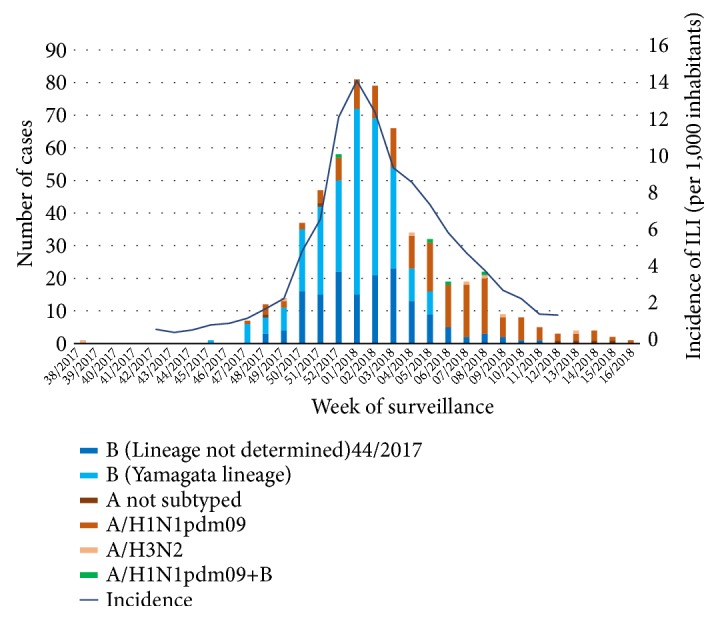
Distribution of samples according to type or subtype of influenza virus identified and incidence of influenza-like illness (ILI) in each week of surveillance in Apulia in the 2017-2018 influenza season.

In Apulia, during the 2017-2018 influenza season, the first two sporadic cases were identified in weeks 38/2017 (A/H3N2) and 45/2017 (B/Yamagata) (Figure 1). The peak of reported ILIs was reached in week 1/2018, with an incidence of 14.32 cases per 1,000 inhabitants. According to the ECDC Moving Epidemic Method [12, 13], the regional incidence returned to the basal level (<2.74 per 1,000 inhabitants) in week 9/2018.

A total of 2,175 patients was tested for influenza viruses, and 565 (26.0%) were positive. IBV strains were identified in 401 of the positive samples (71.0%), with IAV strains in 164 samples (29.0%). At first, most samples contained IBV, but after week 5/2018, identification of A/H1N1pdm09 became predominant (Figure 1). Among the 164 identifications of IAV, 149 (90.8%) were A/H1N1pdm09, 7 (4.3%) were H3N2, and 8 (4.9%) were not subtyped. Four coinfections with A/H1N1pdm09 and IBV were identified. Of the 401 IBV identifications, 269 (67.0%) were characterised and belonged to the Yamagata lineage. The lineage of the other 33.0% of the B viruses was not determined.

**Figure 2:**
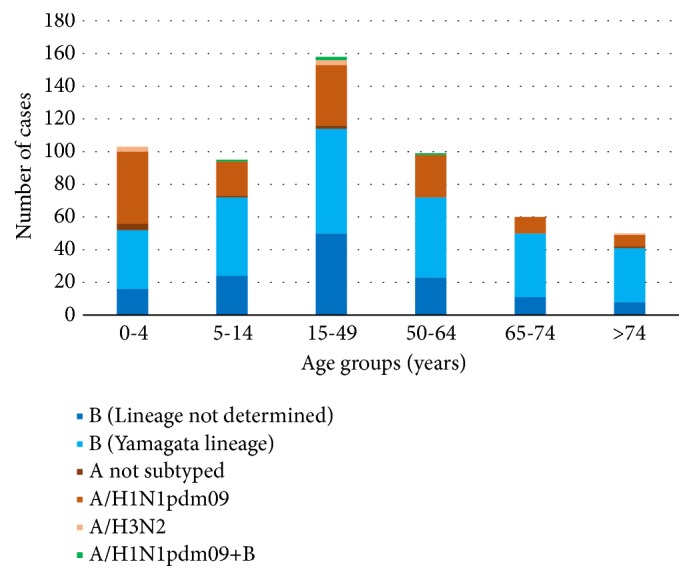
Distribution of influenza-positive cases by age group and type of virus identified, in Apulia in the 2017-2018 influenza season.

Figure 2 shows the distribution of infections by age group and type of virus. In the 0–4-year age group (*n* = 103), IBV was identified in 52 individuals (50.5%), in the 5–14-year age group in 72/95 cases (75.8%), and in the 15–49-year age group in 114/158 cases (72.2%). The prevalence of IBV increased with age, with 72 out of 99 cases (72.7%) in the 50–64-year age group, 50 out of 60 cases (83.3%) in the 65–74-year age group, and 41 out of 50 cases (82.0%) in the >74-year age group testing positive for IBV.

**Figure 3:**
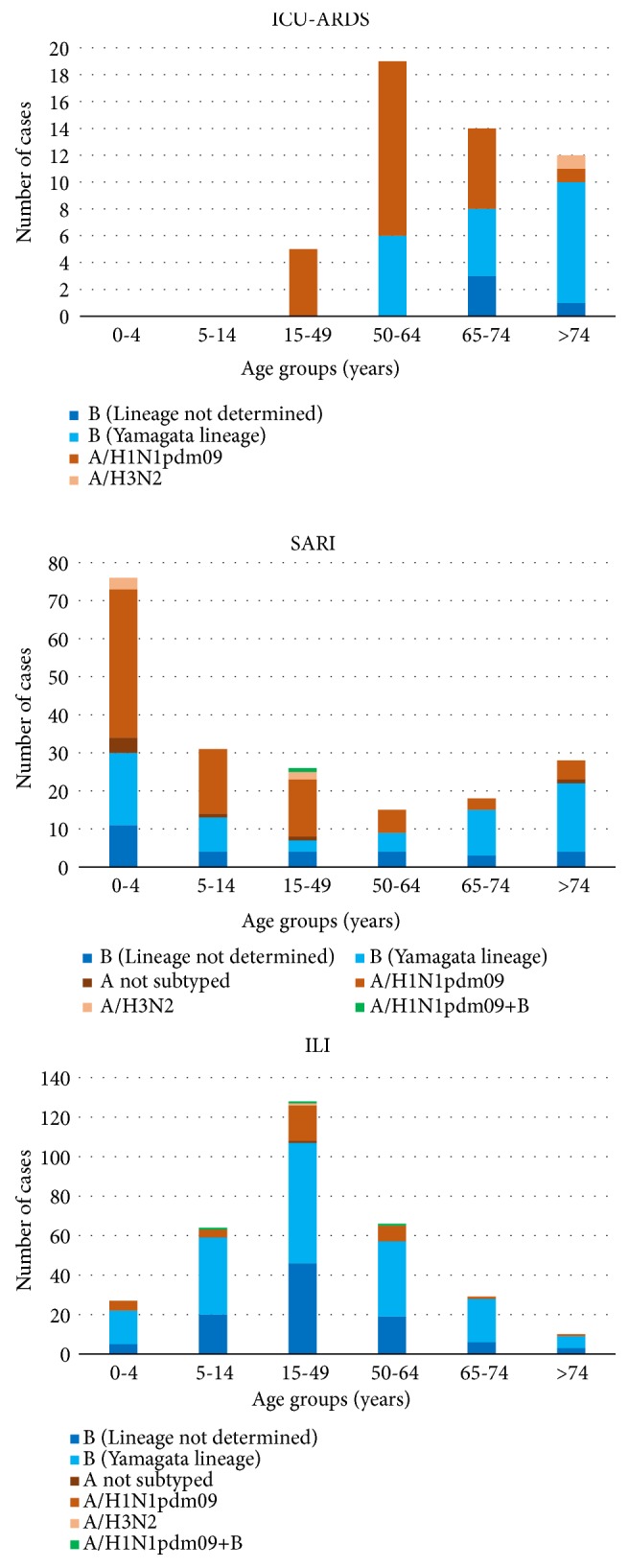
Distribution of the number of cases of influenza infection with ARDS requiring admission to an intensive-care unit (ICU-ARDS), with severe acute respiratory infection (SARI), and with influenza-like illness (ILI-sentinel patients) by age group and type of virus identified, in Apulia in the 2017-2018 influenza season.

ICU-ARDS occurred in 50 individuals, 8 of whom were vaccinated for the 2017-2018 influenza season, and all but 3 of whom had comorbidities. Death occurred in 23 of these individuals (46.0%). The age group with the highest proportion of ICU-ARDS was the >74-year age group, in which 12 of the 50 individuals with influenza (24.0%) had ICU-ARDS, whereas no children <15 years old were affected. Viruses identified in ICU-ARDS were A/H1N1pdm09 (*n* = 25), A/H3N2 (*n* = 1), and IBV (*n* = 24), notably B/Yamagata (*n* = 20).  Figure 3 shows the distribution of the number of cases of influenza by age group and type of virus identified for the ICU-ARDS, SARI, and ILI cases. ICU-ARDS in the 15–49-year and 50–64-year age groups was mostly associated with influenza A/H1N1pdm09 infection, whereas among the 65–74-year and >74-year age groups, most of the ICU-ARDS infections were with IBV. In the >64-year age group, 14 of 18 IBV infections (77.8%) involved B/Yamagata virus. SARI were more frequent in children aged less than 5 years, with the majority of cases associated with IAV, and, in particular, 51.3% were caused by A/H1N1pdm09. In ILI cases, the 15-49-year age group was the most represented and 83.6% were due to B virus.

The comparison of the proportions of IBV cases by age groups showed that IBV infection was significantly more frequent in patients with severe influenza (ICU-ARDS and SARI cases) compared to sentinel patients (ILI cases) for the 0–4-year age group (*p*<0.0001) and >74-year age group (*p*=0.002). For the 5–14, the 15–49, and the 50–64-year age groups, IBV infection was significantly more frequent in ILI cases compared to patients with severe influenza (*p*<0.0001), whereas no significant difference was found for the 65–74-year age group. A/H1N1pdm09 was significantly more frequent in all ICU-ARDS and SARI cases compared to ILI cases (*p*<0.05), except for the 15–49-year age group (*p*>0.05).

**Figure 4:**
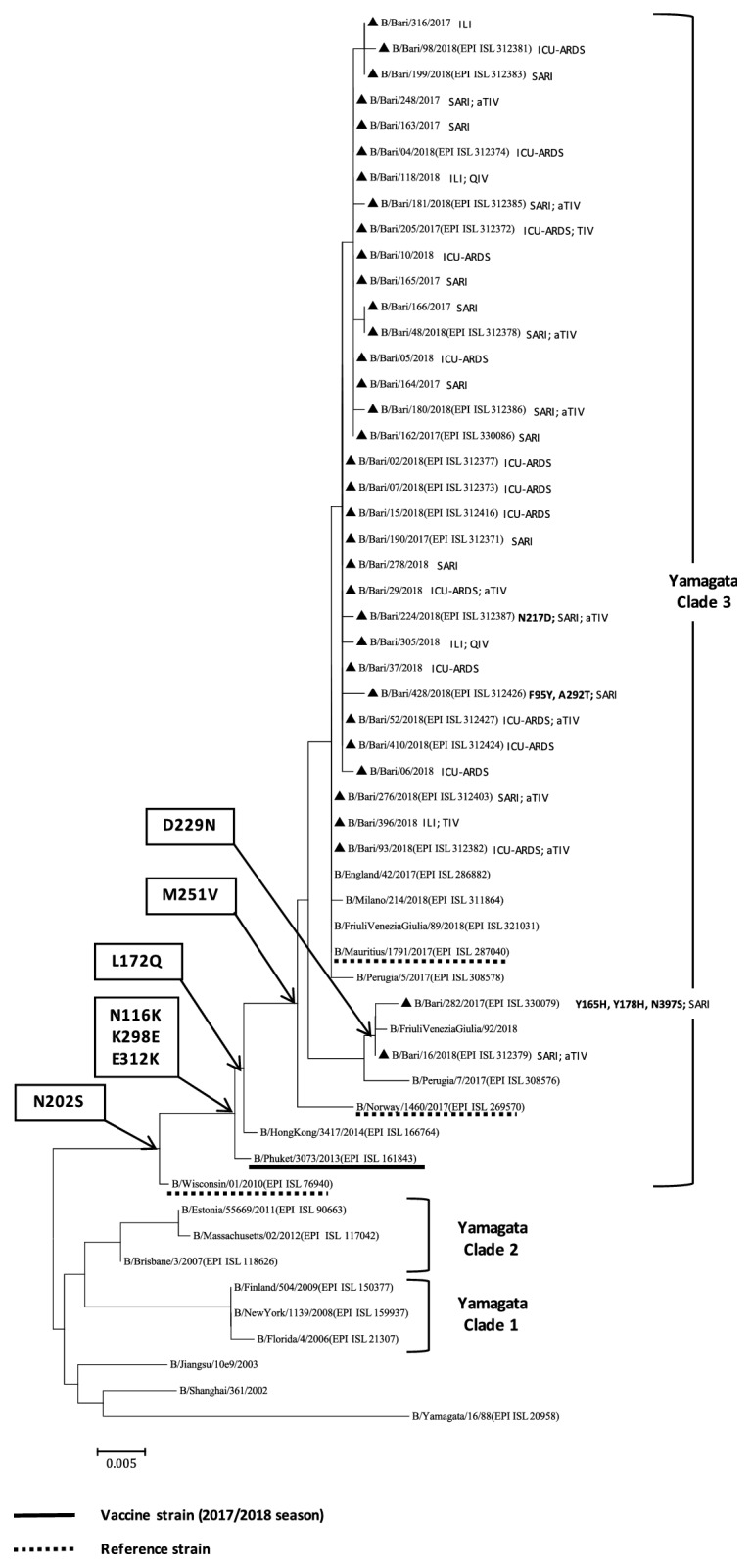
Phylogenetic analysis of the haemagglutinin (HA) gene of 35 B/Yamagata strains characterised in Apulia in the 2017-2018 season (black triangles). The phylogenetic tree was constructed using Mega 7 software, applying the Neighbor Joining algorithm using Kimura-2 parameter-corrected distances. Amino acid substitutions defining specific genetic clusters are shown at nodes. Genetic clades are shown to the right of the tree, and the scale bar represents the nucleotide substitutions per site. For each sequence deposited in GISAID, the accession number is indicated in parentheses. Strain-specific amino acid substitutions are indicated after strain names (in bold font), as are clinical definitions (ILI, influenza-like illness; SARI, severe acute respiratory illness; ICU-ARDS, cases with acute respiratory distress syndrome requiring admission to an intensive-care unit) and, if vaccinated, the type of vaccine (TIV, trivalent vaccine; aTIV, adjuvanted trivalent vaccine; QIV, quadrivalent vaccine).

Phylogenetic analysis of the HA genes of the 35 IBV strains characterised during the 2017-2018 influenza season (Figure 4) showed that all of the B/Yamagata strains clustered into clade 3 (with reference strains B/Wisconsin/01/2010 and B/Phuket/3073/2013, of which the latter was the vaccine strain contained in the quadrivalent vaccine). Among the 35 IBV strains, 14 (40.0%) were identified in vaccinated patients. In particular, two patients, belonging to the 50–64-year age group, were vaccinated with the quadrivalent vaccine (QIV), two with the trivalent vaccine (TIV), and ten with the adjuvanted trivalent vaccine (aTIV). All the strains identified in Apulia had N116K, K298E, and E312K amino acid substitutions, relative to the B/Wisconsin/01/2010 reference strain. In addition, the strains identified in Apulia, in common with most of those identified in Italy, were characterised by L172Q and M251V amino acid substitutions, relative to both of the reference strains. Moreover, two strains characterised in Apulia had the D229N substitution, and one of these strains also had the Y165H, Y178H, and N397S substitutions. In addition to N116K, K298E, E312K, L172Q, and M251V, B/Bari/428/2018 had F95Y and A292T substitutions, and B/Bari/224/2018 had the N217D substitution. No amino acid substitutions within the four major epitope domains comprising the 120-loop (amino acids 116–137), 150-loop (amino acids 141–150), 160-loop (amino acids 162–167), and 190-helix (amino acids 194–202) [14] were identified in the characterised IBVs relative to the vaccine strain. No B/Victoria strains were identified in Apulia.

**Figure 5:**
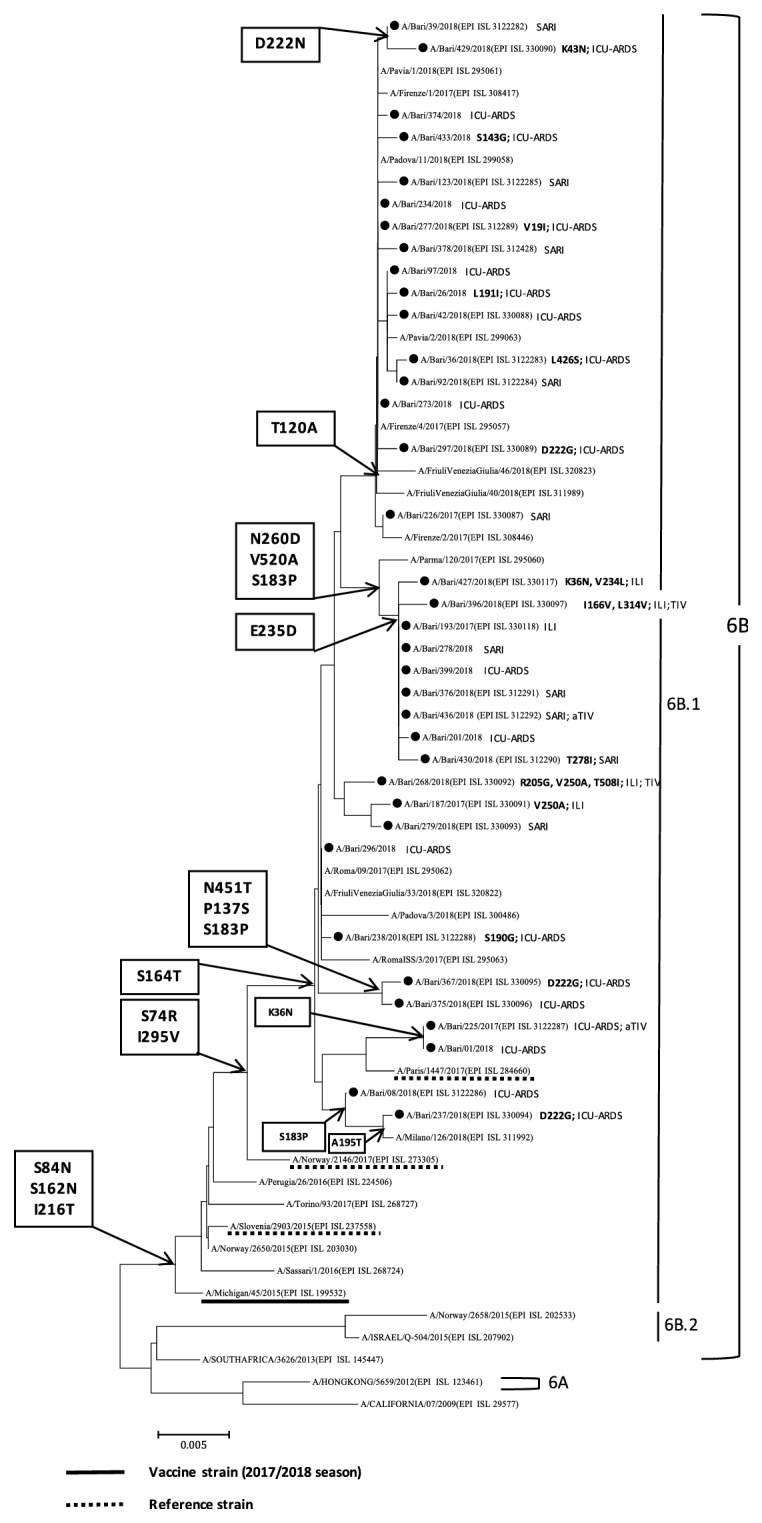
Phylogenetic analysis of the haemagglutinin (HA) gene of the 36 A/H1N1pdm09 strains characterised in Apulia in the 2017-2018 season (black dots). The phylogenetic tree was constructed using Mega 7 software applying the Neighbor Joining algorithm using Kimura-2 parameter-corrected distances. Amino acid substitutions defining specific genetic clusters are shown at nodes. Genetic clades are shown to the right of the tree, and the scale bar represents nucleotide substitutions per site. For each sequence deposited in GISAID, the accession number is indicated in parentheses. Strain-specific amino acid substitutions are indicated after strain names (in bold font), as are clinical definitions (ILI, influenza-like illness; SARI, severe acute respiratory illness; ICU-ARDS, cases with acute respiratory distress syndrome requiring admission to an intensive-care unit) and, if vaccinated, the type of vaccine (TIV, trivalent vaccine; aTIV, adjuvanted trivalent vaccine; QIV, quadrivalent vaccine).

All 36 characterised A/H1N1pdm09 strains belonged to subclade 6B.1 and showed S74R, I295V, and S164T substitutions relative to the reference and vaccine strain A/Michigan/45/2015 (Figure 5). Four strains were identified in vaccinated patients, two of whom were vaccinated with TIV and two with aTIV. Numerous other amino acid substitutions were present in the Apulian A/H1N1pdm09 strains, including T120A in 16 strains and the combination of E235D, S183P, V520A, and N260D in 9 others. Among the 25 Apulian A/H1N1pdm09 ICU-ARDS identifications, 3 strains had the D222G substitution and 1 had D222N, which was also present in one SARI infection.

Phylogenetic analysis of the two A/H3N2 strains characterised in Apulia (data not shown) revealed that one strain clustered with the genetic subgroup 3C.2a (reference vaccine strain Hong Kong/4801/2014) and the other within subclade 3C.2a1 (reference strain A/Singapore/INFMH160019/2016, which is the vaccine strain for the 2018-2019 season in the northern hemisphere).

## 4. Discussion

The monitoring of influenza epidemiology and disease and the characterisation of circulating strain provide data to public-health authorities to facilitate the management of influenza epidemics and the planning of control measures.

In Apulia, as in the rest of Italy, the 2017-2018 influenza season had one of the greatest impacts on public health throughout the past 10 years [8]. In Apulia, the incidence of ILI was similar to that registered overall in Italy, but the peak was reached 1 week earlier [8]. IAV and IBV cocirculated, with a high incidence of IBV in the first part of the season, causing extensive outbreaks in adults and notably in the elderly. After the peak was reached in Apulia, the incidence of IBV infection fell, and infection with A/H1N1pdm09 became predominant. The proportion of influenza infections resulting from IBV was higher in Apulia than in Italy as a whole (71.0% versus 60.0%) [9].

The 2017-2018 influenza season was characterised by an IBV lineage mismatch with the WHO-recommended TIV IBV component (B/Brisbane/60/2008-like virus, Victoria-lineage) [15]. All the IBVs characterised in Apulia belonged to the Yamagata lineage, although in the rest of Italy a few sporadic cases of B/Victoria were also reported [9]. Almost all of the vaccinated patients with IBV infections had received TIV or aTIV, suggesting that the lineage mismatch contributed to the severe impact of the influenza epidemic. Notably, all of the characterised B/Yamagata strains belonged to clade 3, as did B/Phuket/3073/2013, which was contained in the QIV, but not in the TIV or aTIV [15].

The phylogenetic analysis of the HA gene of the A/H1N1pdm09 virus showed that all the Apulian strains belonged to the subclade 6B.1, as did the 2017-2018 vaccine strain. Replacement of the A/California/7/2009(H1N1)pdm09-like 2016–2017 vaccine strain with the A/Michigan/45/2015(H1N1)pdm09 strain in the 2017-2018 formulation increased vaccine effectiveness against A/H1N1pdm09 in Europe to 55–68% [16].

Five A/H1N1pdm09 strains identified in Apulia had D222N or D222G substitutions, and four of these strains were from individuals with ICU-ARDS. These variants, which have been present in Italy since 2009 [17], are associated with clinical severity because of enhancement of the binding affinity for the alpha-2,3 sialic acid receptor (avian-like) in the lower respiratory tract, compared with the affinity for the alpha-2,6 sialic acid receptor that is present at higher density in the upper respiratory tract [18–20]. Binding to the alpha-2,3 sialic acid receptor enables these strains to replicate at high levels in lung tissue, resulting in severe disease [21].

Our data showed that ~70% of ICU-ARDS associated with IBV occurred in individuals >64 years old, which was a similar situation to that in Italy as a whole [22]. The aTIV was recommended in Italy for the 2017-2018 season for this age group and provided free of charge [4]. Because of the impact of IBV in the elderly in the 2017-2018 influenza season, the Italian Ministry of Health has also recommended, for the 2018-2019 season, the use of QIV in individuals aged 65–74 years [23]. In Apulia region, for the elderly, the preferential use of QIV was recommended only for individuals in the 65–74-year age group who were at low risk (without multiple comorbidities) [24].

The use of influenza vaccines in those >64 years old is under debate in response to contrasting evidence. On the one hand, the use of QIV in those aged 65–74 years with no comorbidities has been shown to be associated with substantial health and financial benefits [25], and a switch from aTIV to QIV has been predicted to be most cost-effective for individuals >64 years old [26]. In addition, administration of the QIV induces an immune response that is considered to be protective according to the European Medicines Agency criteria, even in the >64-year age group [27]. On the other hand, evidence has been presented that the use of QIV in the >64-year age group would result in higher costs and lower health benefits than the use of aTIV [28]. An Italian Health Technology Assessment has also indicated that the use of aTIV in the elderly seems to be the best choice [29]. QIV may not result in a significant advantage in the elderly because aTIV can produce substantial cross-protection between the two linages of IBV, independent of the match between the circulating strain and the vaccine strain [30–34]. Further evidence of the relative effectiveness of the different influenza vaccines in the elderly is needed for definitive recommendations to be made.

This study may have some limitations. First, a bias could have occurred because the strains selected for the sequence analyses were mostly from patients with SARI and ICU-ARDS and may not have been fully representative of the circulating strains in Apulia. Second, we have no data on the possible circulation of strains that were resistant to antiviral therapy, because the NA gene was not sequenced.

## 5. Conclusions

In conclusion, our data provide information on the most recent influenza season and on the molecular epidemiology of the circulating influenza viruses in Apulia, a densely populated region of Italy. Continuous epidemiological and virological surveillance remain an essential tool for monitoring of the influenza season and the characteristics of circulating viruses, to identify possible mismatches with seasonal vaccine strains and provide information to improve vaccination strategies.

## Data Availability

The data that support the findings of this study are available from the corresponding author on reasonable request.

## Abbreviations

ARDS: Acute respiratory distress syndrome
ARI: Acute respiratory infection
aTIV: Adjuvanted trivalent influenza vaccine
IAV: Influenza A virus
IBV: Influenza B virus
ILI: Influenza-like illness
ICU-ARDS: Case of influenza infection with ARDS requiring admission to an intensive-care unit
QIV: Quadrivalent influenza vaccine
SARI: Severe acute respiratory infection
TIV: Trivalent influenza vaccine.
